# M-Polynomials and Degree-Based Topological Indices of the Crystallographic Structure of Molecules

**DOI:** 10.3390/biom8040107

**Published:** 2018-10-03

**Authors:** Wei Gao, Muhammad Younas, Adeel Farooq, Abid Mahboob, Waqas Nazeer

**Affiliations:** 1 School of Information Science and Technology, Yunnan Normal University, Kunming 650500, China; gaowei@ynnu.edu.cn; 2 Department of Mathematics, COMSATS University Islamabad, Lahore Campus, Lahore 54000, Pakistan; muhammadyounas@cuilahore.edu.pk (M.Y.); adeelfarooq@cuilahore.edu.pk (A.F.); 3 Department of Mathematics, University of Education, Vehari Campus, Lahore 54000, Pakistan; abid.mahboob@ue.edu.pk; 4 Division of Science and Technology, University of Education, Lahore 54000, Pakistan

**Keywords:** M-polynomial, zagreb index, randić index, crystallographic structure

## Abstract

Topological indices are numerical parameters used to study the physical and chemical properties of compounds. In quantitative structure–activity relationship QSARs, topological indices correlate the biological activity of compounds with their physical properties like boiling point, stability, melting point, distortion, and strain energy etc. In this paper, we determined the M-polynomials of the crystallographic structure of the molecules Cu_2_O and T_i_F_2_ [*p,q,r*]. Then we derived closed formulas for some well-known topological indices using calculus. In the end, we used Maple 15 to plot surfaces associated with the topological indices of Cu_2_O and T_i_F_2_ [*p,q,r*].

## 1. Introduction

In the medication mathematical model, the structure of medication is taken as an undirected graph, where vertices and edges are taken as atoms and chemical bonds. With the rapid advancement of medicine manufacture, a huge number of new medications are created every year. Henceforth, it requires a colossal amount of work to decide on the pharmacological, compound and biological characteristics of these new medications, and such workloads turn out to be increasingly particular and bunched together. It requires sufficient reagents equipment and partners to test the performances and the reactions of new medications. Be that as it may, in run down poor nations and regions, (for example, certain urban communities and nations in South America, Southeast Asia, Africa and India), there is no adequate cash to acquire reagents and equipment which can be utilized to measure the biochemical properties.

Luckily, numerous past studies [1,2] have pointed out that chemical and pharma codynamic attributes of medications and their atomic structures are firmly connected. In the event that we compute indicators of these graphs of drug molecules [3,4,5,6] with the perspective of characterizing the topological index, pharmaceutical researchers could well think that it is helpful to understand the medicinal properties, which can make up for the deformities of drug and chemical experiments. From this outlook, the strategies on topological index calculation are exceptionally appropriate and functional for developing nations where they can yield the available biological and medical information of new medications without compound investigation equipment and experiment.

In spite of the fact that there have been a few contributions on distance-based indices and degree-based indices, [6,7,8,9] the investigations of the topological index for certain uncommon structures are still to a great extent restricted. As a result of this, enormous scholarly and modern thinking has been pulled in to examine the topological indices of drug structures from a mathematical perspective.

Mathematical chemistry gives tools, for example, polynomials and numbers to obtain properties of chemical compounds without utilizing quantum mechanics. A topological index is a numerical parameter of a graph and describes its topology. It depicts the molecular structure numerically and is utilized in the advancement of qualitative structure activity relationships (QSARs). There are three kinds of topological indices:

1. degree-based.
2. distance-based.
3. spectral-based.

Degree-based topological indices have been studied extensively and can be correlated with many properties of the understudy molecular compounds. There is a strong relationship between distance-based and degree-based topological indices [10]. Topological indices are really the numerical values that relate the structure to different physical properties, synthetic reactivity, and organic biological activities [11,12,13,14,15]. Many properties, for example, heat of formation, boiling point, strain energy, rigidity, and fracture toughness of a molecule are strongly connected to its molecular graph.

Hosoya polynomial, (Wiener polynomial), [16] assumes an essential part in distance based topological indices. A considerable rundown of distance based indices can be effectively assessed from the Hosoya polynomial. A comparable leap forward was acquired as of late by Deutsch and Klavžar [17], with regards to degree-based indices. They presented M-polynomial in 2015 to assume a part, parallel to Hosoya polynomial, to decide on the closed form of numerous degree-based topological indices [18,19,20,21,22]. The genuine intensity of the M-polynomial is its extensive nature containing solid data about degree-based graph invariants. These invariants are graph based on symmetries displayed in the 2d-atomic grids and altogether explain a few properties of the material under perception.

A lot of research has been done in the direction of M-polynomial, for example Munir et al. computed M-polynomial and related indices of triangular boron nanotubes in [21], polyhex-nanotubes in [22], nanostar dendrimers in [18], titania nanotubes in [19], as well as M-Polynomials and topological indices of V-Phenylenic Nanotubes and Nanotori in [20]. In this paper we aim to compute the M-polynomial of the crystallographic structure of the molecule Cu_2_O and the crystal structure of titanium difluoride T_i_F_2_ [*n,m,t*]. We also recover the first and second Zagreb indices, the modified Zagreb index, the Symmetric division index, the Harmonic index, the Randić and the Inverse Randić index, the Augmented Zagreb index and the Inverse sum index for these molecules. We also plot our results to determine the dependence on the involved parameters. For more details about the topological indices and their applications, we refer to references [23,24,25,26,27].

## 2. Basic Definitions and Literature Review

Throughout this article, we assume *G* to be a simple connected graph, *V (G)* and *E (G)* are the vertex set and the edge set respectively and *d_v_* denotes the degree of a vertex *v*.

**Definition** **1.** *The M-polynomial [17] of G is defined as:* $M\;\left(G,x,y\right)=\sum_{\delta \leq i\leq j\leq \Delta }m_{ij}\left(G\right)x^{i}y^{j}$ *where* $\delta =\mathrm{Min}\{d_{v}|v\in V\;(G)\},$ $\Delta =\mathrm{Max}\{d_{v}|v\in V\;(G)\},$ *and* $m_{ij}(G)$ *is the edge* vu∈E(G) *such that where* i≤j *.*

Wiener index [28] is the first topological index and its various applications are discussed in [29,30]. Randić index, $R_{-1/2}(G)$, is introduced by Milan Randić in 1975 defined as: $R_{-1/2}(G)=\sum_{uv\in E(G)}\frac{1}{\sqrt{d_{u}d_{v}}}.$ For general details about $R_{-1/2}(G)$ and its generalized Randić index, $R_{\alpha }(G)=\sum_{uv\in E(G)}\frac{1}{{\left(d_{u}d_{v}\right)}^{\alpha }},$ please see [31,32,33,34,35].

The inverse Randić index is defined as $RR_{\alpha }(G)=\sum_{uv\in E(G)}{\left(d_{u}d_{v}\right)}^{\alpha }.$ Clearly $R_{-1/2}(G)$ is a special case of $R_{\alpha }(G)$ when $\alpha =-\frac{1}{2}$. This index has many applications in diverse areas. Many papers and books such as [36,37,38] are written on this topological index as well. Gutman and Trinajstić introduced two indices namely the first Zagreb index and the second Zagreb index and are defined as: $M_{1}(G)=\sum_{uv\in E(G)}\left(d_{u}+d_{v}\right)$ and $M_{2}(G)=\sum_{uv\in E(G)}\left(d_{u}\times d_{v}\right)$. The second modified Zagreb index is defined as: ${}^{m}M_{2}\left(G\right)\;=\sum_{uv\in E(G)}\frac{1}{d(u)d(v)}.$ We refer [39,40,41,42,43,44,45,46,47,48] to the readers for comprehensive details of these indices. Other famous indices are the Symmetric division index: SDD(G) = $\sum_{uv\in E(G)}\left\{\frac{\min(d_{u},d_{v})}{\max(d_{u},d_{v})}+\frac{\max(d_{u},d_{v})}{\min(d_{u},d_{v})}\right\}$, the Harmonic index: $H(G)=\sum_{vu\in E(G)}\frac{2}{d_{u}+d_{v}}.$ Inverse sum index: $I(G)=\sum_{vu\in E(G)}\frac{d_{u}d_{v}}{d_{u}+d_{v}}$ and augmented Zagreb index: $A(G)=\sum_{vu\in E(G)}{\left\{\frac{d_{u}d_{v}}{d_{u}+d_{v}-2}\right\}}^{3},$ [49,50].

Table 1 presented in [7,8,9] relates some of these well-known degree-based topological indices with the M-polynomial with the following reserved notations

(1) $$\begin{array}{l} D_{x}=x\frac{\partial \left(f\left(x,y\right)\right)}{\partial x},\;D_{y}=y\frac{\partial \left(f\left(x,y\right)\right)}{\partial y},\;S_{x}=\overset{x}{\underset{0}{\int }}\frac{f\left(t,y\right)}{t}dt,S_{y}=\overset{y}{\underset{0}{\int }}\frac{f\left(x,t\right)}{t}dt,\\ J\left(f\left(x,y\right)\right)=f\left(x,x\right),\;Q_{\alpha }\left(f\left(x,y\right)\right)=x^{\alpha }f\left(x,y\right). \end{array}$$

## 3. Methodology

First of all we associated the graphs with Cu_2_O and T_i_F_2_ [*p,q,r*] where atoms are represented by vertices and chemical bonds are represented by edges. Then by using the symmetry of the molecular structures of Cu_2_O and T_i_F_2_ [*p,q,r*] we counted the edges and vertices by a simple counting method. By applying the formula of the polynomial we derived the M-polynomials of Cu_2_O and T_i_F_2_ [*p,q,r*]. From these M-polynomials we recovered nine degree-based topological indices by using calculus. We used Maple 2015 to plot our results.

## 4. Main Results

### 4.1. M-Polynomial and Degree-Based Topological Indices of Cu_2_O

**Figure 1:**
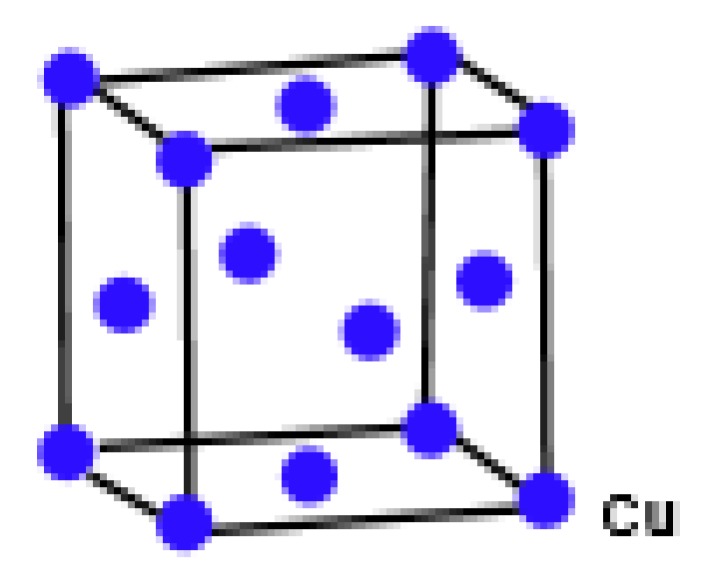
Crystallographic structure of Cu_2_O.

**Figure 2:**
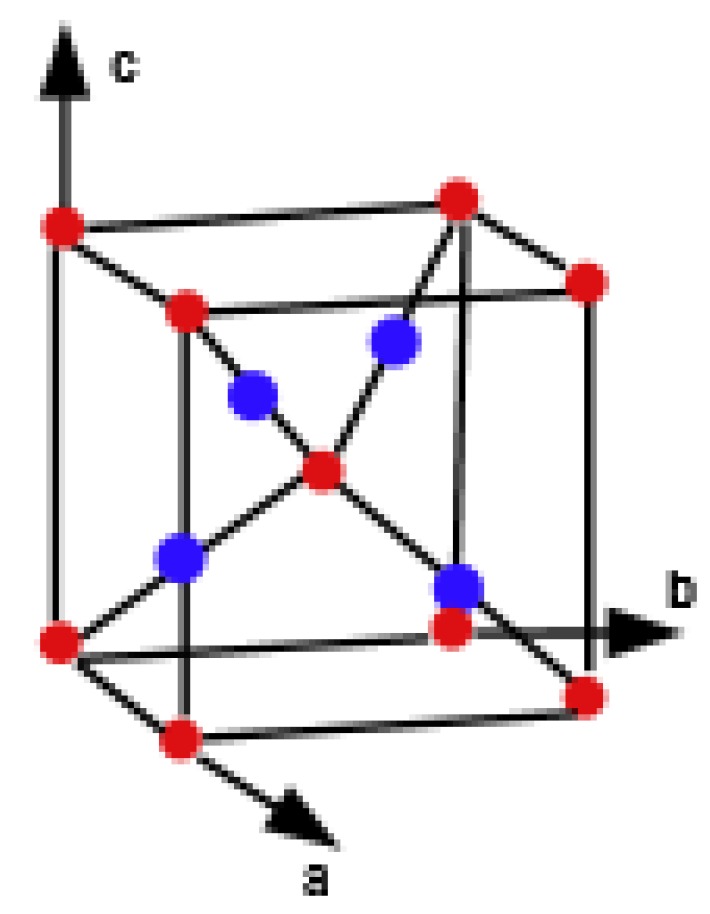
Formation of Cu_2_O lattices.

**Figure 3:**
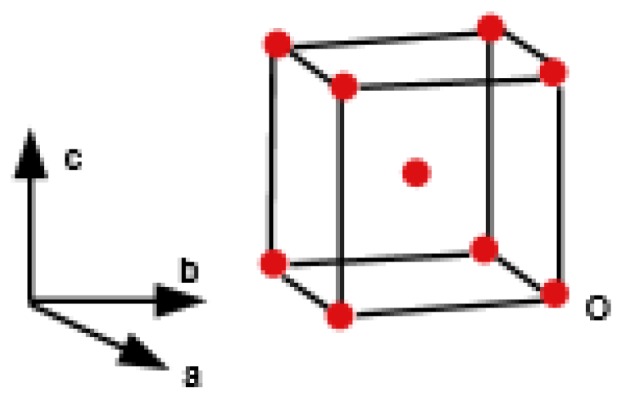
Unit cell of Cu_2_O.

**Figure 4:**
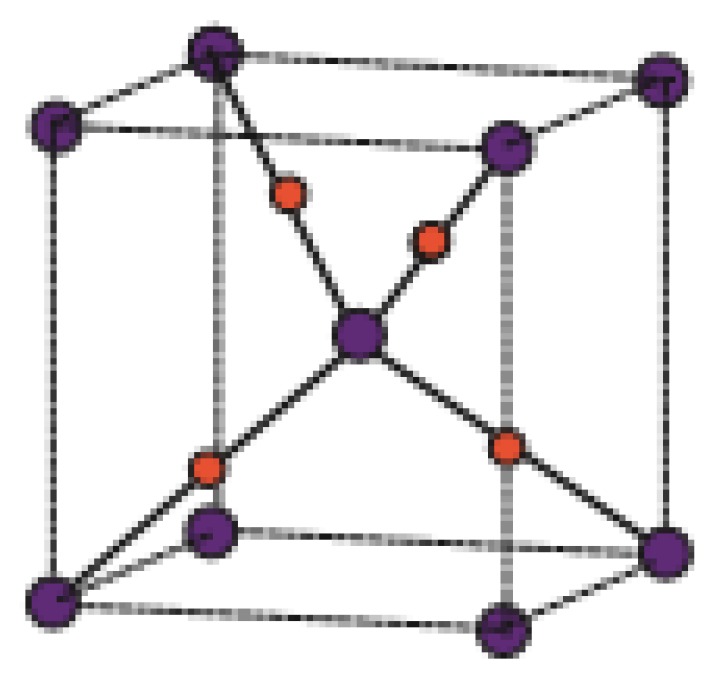
Cu_2_O[1,1,1].

**Figure 5:**
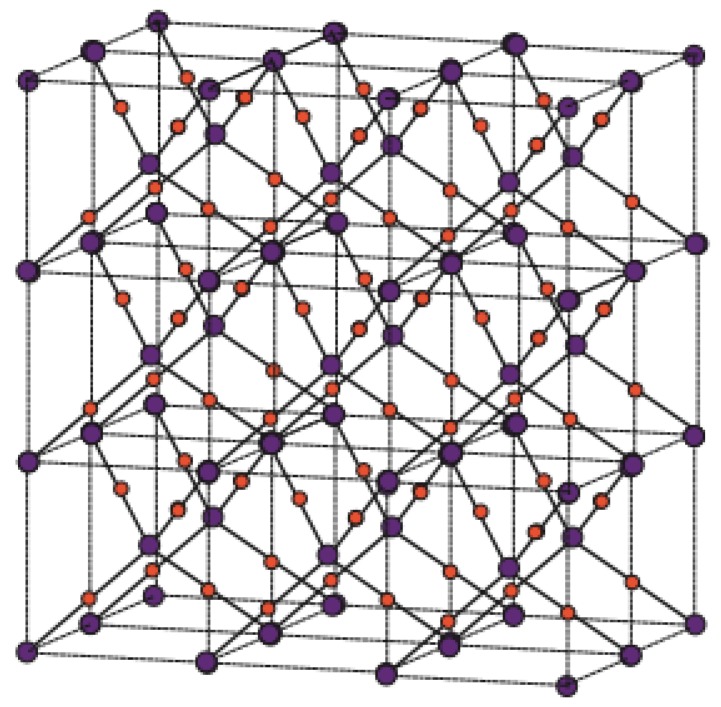
Cu_2_O[3,2,3].

Copper(I) oxide or cuprous oxide is the inorganic compound with the formula Cu_2_O. It is one of the principal oxides of copper, the other being CuO or cupric oxide. The solid is diamagnetic. In terms of their coordination spheres, copper centers are 2-coordinated and the oxides are tetrahedral. The structure thus resembles in some sense the main polymorphs of SiO_2_, and both structures feature interpenetrated lattices. Copper(I) oxide dissolves in concentrated ammonia solution to form the colorless complex [Cu(NH_3_)_2_]^+^, which is easily oxidized in air to the blue [Cu(NH_3_)_4_(H_2_O)_2_]^2+^. It dissolves in hydrochloric acid to give solutions of CuCl_2_^−^. Dilute sulfuric acid and nitric acid produce copper(II) sulfate and copper(II) nitrate, respectively. Cuprous oxide is commonly used as a pigment, a fungicide, and an antifouling agent for marine paints. Rectifier diodes based on this material have been used industrially as early as 1924, long before silicon became the standard. Copper(I) oxide is also responsible for the pink color in a positive Benedict’s test. This is the main reason to choose Cu_2_O. Nowadays the Crystallographic Structure of the Molecule Cu_2_O has attracted attention due to its interesting properties, low-cost, abundance, non-toxic nature, and simple fabrication process [51]. The promising applications of Cu_2_O mainly focus on chemical sensors, solar cells, photocatalysis, lithium-ion batteries, and catalysis [52]. Figure 1, Figure 2, Figure 3, Figure 4 and Figure 5 describe the graph of the molecule Cu_2_O [53]. The crystallographic structure of Cu_2_O is shown in Figure 1. In the lattice of Cu_2_O the structural characteristics of the atoms of O and C_u_ are shown in Figure 2. By interpenetrating the O lattices with Cu lattices, Cu_2_O lattices are formed. The unit cell of Cu_2_O is shown in Figure 3. In Figure 3 copper atoms are shown by red dots and oxygen atoms are shown by blue dots. In the Cu_2_O lattice graph, each copper atom is attached to two oxygen atoms, and every oxygen atom is attached to four copper atoms. Cu_2_O [1,1,1] and Cu_2_O [3,2,3] are shown in Figure 4 and Figure 5 respectively.

Let *G* ≅ Cu_2_O[*p,q,r*] be the chemical graph of Cu_2_O with *p × q* unit cells in the plane and r layers [14]. It can be observed that

$$\left|V\left(Cu_{2}O[p,q,r]\right)\right|=6prrt+pq+qr+pr+q+p+r+1\;\left|E\left(Cu_{2}O[p,q,r]\right)\right|=8pqr.$$

**Theorem** **1.** *For the graph of crystallographic structure G* ≅ Cu_2_O[*p,q,r*]*, we have*

$$\begin{array}{ll} M\left(G,\;x,y\right)= & 4\left(p+q+r-2\right)xy^{2}+4\left(pq+pr+qr-2\left(p+q+r\right)+3\right)x^{2}y^{2}\\ & +4\left(2pqr-\left(pq+pr+qr\right)+p+q+r-1\right)x^{2}y^{4}. \end{array}$$

**Proof.**  Let *G* be the crystallographic structure of Cu_2_O[*p,q,r*]. The edge set of Cu_2_O[*p,q,r*] has the following three partitions,

$$E_{1}=E_{\left\{1,2\right\}}=\left\{e=uv\in E\left(G\right)|d_{u}=1,d_{v}=2\right\},$$

$$E_{2}=E_{\left\{2,2\right\}}=\left\{e=uv\in E\left(G\right)|d_{u}=2,\;d_{v}=2\right\},\;E_{3}=E_{\left\{2,4\right\}}=\left\{e=uv\in E\left(G\right)|d_{u}=2,\;d_{v}=4\right\},$$

such that

$$\left|E_{1}\left(G\right)\right|=4p+4q+4r-8,$$

$$\left|E_{2}\left(G\right)\right|=4pq+4pr+4qr-8p-8q-8r+12.\;\left|E_{3}\left(G\right)\right|=4(2pqr-pq-pr-qr+p+q+r-1).$$

Thus the M-polynomial of Cu_2_O[*n,m,t*] is

$$\begin{array}{ll} M\left(G;x,y\right) & =\underset{i\leq j}{\sum }m_{ij}\left(G\right)x^{i}y^{j}\\ & =\underset{1\leq 2}{\sum }m_{12}\left(G\right)xy^{2}+\underset{2\leq 2}{\sum }m_{22}\left(G\right)x^{2}y^{2}+\sum_{2\leq 4}m_{24}\left(G\right)x^{2}y^{4}\\ & =\underset{uv\in E_{1}}{\sum }m_{12}\left(G\right)xy^{2}+\underset{uv\in E_{2}}{\sum }m_{22}\left(G\right)x^{2}y^{2}+\underset{uv\in E_{3}}{\sum }m_{24}\left(G\right)x^{2}y^{4}\\ & =\left|E_{1}\left(G\right)\right|xy^{2}+\left|E_{2}\left(G\right)\right|x^{2}y^{2}+\left|E_{3}\left(G\right)\right|x^{2}y^{4}\\ & =\left(4p+4q+4r-8\right)xy^{2}+\left(4pq+4pr+4qr-8p-8q-8r+12\right)x^{2}y^{2}\\ & \;+4\left(2pqr-pq-pr-qr+p+q+r-1\right)x^{2}y^{4}.\\ & =4\left(p+q+r-2\right)xy^{2}+4\left(pq+pr+qr-2\left(p+q+r\right)+3\right)x^{2}y^{2}\\ & \;+4\left(2pqr-\left(pq+pr+qr\right)+p+q+r-1\right)x^{2}y^{4}. \end{array}$$

☐

**Theorem** **2.** *For the graph of the crystallographic structure G* = Cu_2_O[*p,q,r*]*, we have*

$$M_{1}\left(G\right)=48pqr-8\left(pq+pr+qr\right)+4\left(p+q+r\right).$$

**Proof.**  Let

$$\begin{array}{ll} M\left(G,\;x,y\right)= & f(x,y)=4\left(p+q+r-2\right)xy^{2}+4\left(pq+pr+qr-2\left(p+q+r\right)+3\right)x^{2}y^{2}\\ & +4\left(2pqr-\left(pq+pr+qr\right)+p+q+r-1\right)x^{2}y^{4}. \end{array}$$

Then

(2) $$\begin{array}{ll} D_{x}f(x,y)= & 4\left(p+q+r-2\right)xy^{2}+8\left(pq+pr+qr-2\left(p+q+r\right)+3\right)x^{2}y^{2}\\ & +8\left(2pqr-\left(pq+pr+qr\right)+p+q+r-1\right)x^{2}y^{4}, \end{array}$$

(3) $$\begin{array}{ll} D_{y}f(x,y)= & 8\left(p+q+r-2\right)xy^{2}+8\left(pq+pr+qr-2\left(p+q+r\right)+3\right)x^{2}y^{2}\\ & +16\left(2pqr-\left(pq+pr+qr\right)+p+q+r-1\right)x^{2}y^{4}, \end{array}$$

From Table 1, Equations (2) and (3)

$$\begin{array}{ll} M_{1}\left(G\right) & ={\left(D_{x}+D_{y}\right)f(x,y)|}_{x=y=1}\\ & =48pqr-8\left(pq+pr+qr\right)+4\left(p+q+r\right). \end{array}$$

**Figure 6:**
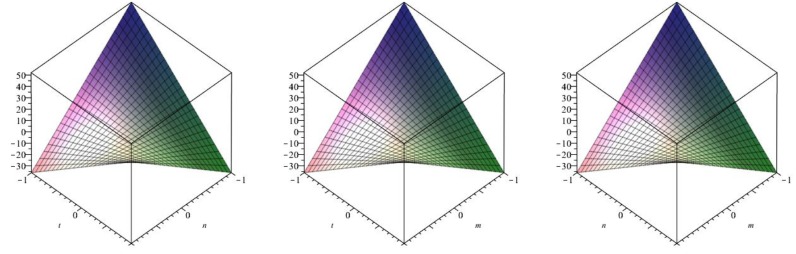
3D plot of first Zagreb index.

The following Figure 6 is a 3D plot of the first Zagreb index (for *p* = 1 left, *q* = 1 middle and *r* = 1 right) and tells us the dependence of the first Zagreb index on the involved parameters. ☐

**Theorem** **3.** *For the graph of the crystallographic structure G* = Cu_2_O[*p,q,r*]*, we have*

$$M_{2}\left(G\right)=64pqr-16\left(pq+pr+qr\right)+8\left(p+q+r\right).$$

**Proof.**  Let

$$\begin{array}{ll} M\left(G,\;x,y\right)= & 4\left(p+q+r-2\right)xy^{2}+4\left(pq+pr+qr-2\left(p+q+r\right)+3\right)x^{2}y^{2}\\ & +4\left(2pqr-\left(pq+pr+qr\right)+p+q+r-1\right)x^{2}y^{4}. \end{array}$$

Then

(4) $$\begin{array}{ll} D_{y}D_{x}f(x,y) & =8\left(p+q+r-2\right)xy^{2}+16\left(pq+pr+qr-2\left(p+q+r\right)+3\right)x^{2}y^{2}\\ & +32\left(2pqr-\left(pq+pr+qr\right)+p+q+r-1\right)x^{2}y^{4}, \end{array}$$

Now from Table 1 and Equation (4)

$$\begin{array}{ll} M_{2}\left(G\right) & ={D_{y}D_{x}(f(x,y))|}_{x=y=1}\\ & =64pqr-16\left(pq+pq+qr\right)+8\left(p+q+r\right). \end{array}$$

**Figure 7:**
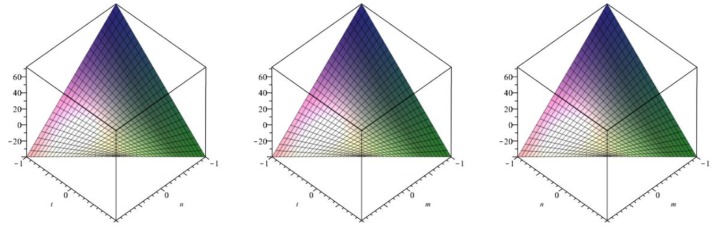
3D plot of second Zagreb index.

The following Figure 7 is a 3D plot of the second Zagreb index (for *p* = 1 left, *q* = 1 middle and *r* = 1 right) and tells us the dependence of the second Zagreb index on the involved parameters. ☐

**Theorem** **4.** *For the graph of the crystallographic structure G* = Cu_2_O[*p,q,r*]*, we have*

M2m(G)=pqr+12(pq+pr+qr)+12(p+q+r)−32.

**Proof.**  Let

$$\begin{array}{ll} M\left(G,\;x,y\right)= & 4\left(p+q+r-2\right)xy^{2}+4\left(pq+pr+qr-2\left(p+q+r\right)+3\right)x^{2}y^{2}\\ & +4\left(2pqr-\left(pq+pr+qr\right)+p+q+r-1\right)x^{2}y^{4}. \end{array}$$

Then

(5) $$\begin{array}{ll} S_{x}S_{y}(f(x,y))= & 2\left(p+q+r-2\right)xy^{2}+\left(pq+pr+qr-2\left(p+q+r\right)+3\right)x^{2}y^{2}\\ & +\frac{1}{2}\left(2pqr-\left(pq+pr+qr\right)+p+q+r-1\right)x^{2}y^{4}, \end{array}$$

Now from Table 1 and Equation (5)

$$\begin{array}{ll} {}^{m}M_{2}\left(G\right) & ={S_{x}S_{y}(f(x,y))|}_{x=y=1}\\ & =pqr+\frac{1}{2}\left(pq+pr+qr\right)+\frac{1}{2}\left(p+q+r\right)-\frac{3}{2}. \end{array}$$

**Figure 8:**
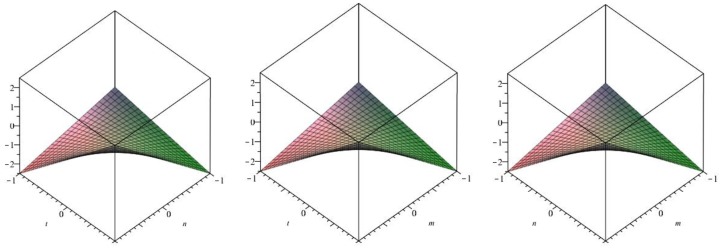
3D plot of modified second Zagreb index.

The following Figure 8 is a 3D plot of the modified second Zagreb index (for *p* = 1 left, *q* = 1 middle and *r* = 1 right) and tells us the dependence of the modified second Zagreb index on the involved parameters. ☐

**Theorem** **5.** *For the graph of the crystallographic structure G* = Cu_2_O[*p,q,r*]*, we have*

$$R_{\alpha }\left(G\right)=2^{3\alpha +3}pqr+\left(2^{2\alpha +2}-2^{3\alpha +2}\right)\left(pq+pr+qr\right)+\left(2^{\alpha +2}-2^{2\alpha +3}+2^{3\alpha +2}\right)\left(p+q+r\right)-\left(2^{\alpha +3}-3\cdot 2^{2\alpha +2}+2^{3\alpha +2}\right).$$

**Proof.**  Let

$$\begin{array}{ll} M\left(G,\;x,y\right)= & 4\left(p+q+r-2\right)xy^{2}+4\left(pq+pr+qr-2\left(p+q+r\right)+3\right)x^{2}y^{2}\\ & +4\left(2pqr-\left(pq+pr+qr\right)+p+q+r-1\right)x^{2}y^{4}. \end{array}$$

Then

(6) $$\begin{array}{ll} D_{x}{}^{\alpha }D_{y}{}^{\alpha }(f(x,y))= & 2^{\alpha +2}\left(p+q+r-2\right)xy^{2}+2^{2\alpha +2}\left(pq+pr+qr-2\left(p+q+r\right)+3\right)x^{2}y^{2}\\ & +2^{3\alpha +2}\left(2pqr-\left(pq+pr+qr\right)+p+q+r-1\right)x^{2}y^{4}, \end{array}$$

Now from Table 1 and Equation (6)

$$\begin{array}{l} R_{\alpha }\left(G\right)={D_{x}^{\alpha }D_{y}^{\alpha }(f(x,y))|}_{x=y=1}\\ =2^{3\alpha +3}pqr+\left(2^{2\alpha +2}-2^{3\alpha +2}\right)\left(pq+pr+qr\right)+\left(2^{\alpha +2}-2^{2\alpha +3}+2^{3\alpha +2}\right)\left(p+q+r\right)-\left(2^{\alpha +3}-3\cdot 2^{2\alpha +2}+2^{3\alpha +2}\right). \end{array}$$

**Figure 9:**
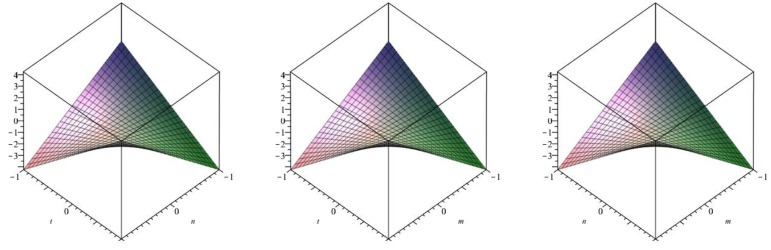
3D plot of Randić index.

The following Figure 9 is a 3D plot of the Randić index (for *p* = 1 left, *q* = 1 middle and *r* = 1 right) and tells us the dependence of the Randić index on the involved parameters. ☐

**Theorem** **6.** *For the graph of the crystallographic structure G* = Cu_2_O[*p,q,r*]*, we have*

$$RR_{\alpha }\left(G\right)=\frac{1}{2^{3\alpha -3}}pqr+\left(\frac{1}{2^{2\alpha -2}}-\frac{1}{2^{3\alpha -2}}\right)\left(pq+pr+qr\right)+\left(\frac{1}{2^{\alpha -2}}-\frac{1}{2^{2\alpha -3}}+\frac{1}{2^{3\alpha -2}}\right)\left(p+q+r\right)-\left(\frac{1}{2^{\alpha -3}}-\frac{3}{2^{2\alpha -2}}+\frac{1}{2^{3\alpha -2}}\right).$$

**Proof.**  Let

$$\begin{array}{ll} M\left(G,\;x,y\right)= & 4\left(p+q+r-2\right)xy^{2}+4\left(pq+pr+qr-2\left(p+q+r\right)+3\right)x^{2}y^{2}\\ & +4\left(2pqr-\left(pq+pr+qr\right)+p+q+r-1\right)x^{2}y^{4}. \end{array}$$

Then

(7) $$\begin{array}{ll} S_{x}{}^{\alpha }S_{y}{}^{\alpha }(f(x,y))= & \frac{1}{2^{\alpha -2}}\left(p+q+r-2\right)xy^{2}+\frac{1}{2^{2\alpha -2}}\left(pq+pr+qr-2\left(p+q+r\right)+3\right)x^{2}y^{2}\\ & +\frac{1}{2^{3\alpha -2}}\left(2pqr-\left(pq+pr+qr\right)+p+q+r-1\right)x^{2}y^{4}, \end{array}$$

Now from Table 1 and Equation (7)

$$\begin{array}{l} RR_{\alpha }\left(G\right)={S_{x}^{\alpha }S_{y}^{\alpha }(f(x,y))|}_{x=y=1}\\ =\frac{1}{2^{3\alpha -3}}pqr+\left(\frac{1}{2^{2\alpha -2}}-\frac{1}{2^{3\alpha -2}}\right)\left(pq+pr+qr\right)+\left(\frac{1}{2^{\alpha -2}}-\frac{1}{2^{2\alpha -3}}+\frac{1}{2^{3\alpha -2}}\right)\left(p+q+r\right)-\left(\frac{1}{2^{\alpha -3}}-\frac{3}{2^{2\alpha -2}}+\frac{1}{2^{3\alpha -2}}\right). \end{array}$$

**Figure 10:**
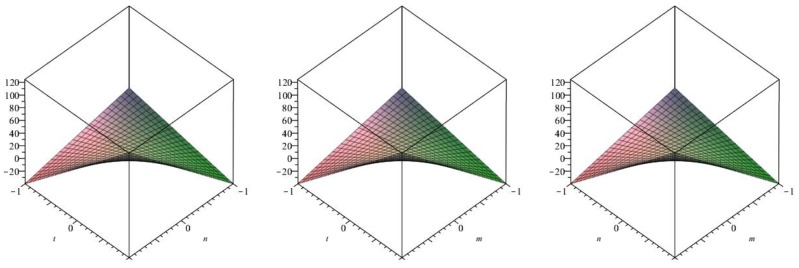
3D plot of Inverse Randić index.

The following Figure 10 is a 3D plot of the inverse Randić index (for *p* = 1 left, *q* = 1 middle and *r* = 1 right) and tells us the dependence of the inverse Randić index on the involved parameters. ☐

**Theorem** **7.** *For the graph of the crystallographic structure G* = Cu_2_O[*p,q,r*]*, we have*

SSD(G)=20pqr−2(pq+pr+qr)+4(p+q+r)−6.

**Proof.**  Let

$$\begin{array}{ll} M\left(G,\;x,y\right)= & 4\left(p+q+r-2\right)xy^{2}+4\left(pq+pr+qr-2\left(p+q+r\right)+3\right)x^{2}y^{2}\\ & +4\left(2pqr-\left(pq+pr+qr\right)+p+q+r-1\right)x^{2}y^{4}. \end{array}$$

Then

(8) $$\begin{array}{ll} S_{y}D_{x}\left(f(x,y)\right)= & 2\left(p+q+r-2\right)xy^{2}+4\left(pq+pr+qr-2\left(p+q+r\right)+3\right)x^{2}y^{2}\\ & +2\left(2pqr-\left(pq+pr+qr\right)+p+q+r-1\right)x^{2}y^{4}, \end{array}$$

(9) $$\begin{array}{ll} S_{x}D_{y}\left(f(x,y)\right)= & 8\left(p+q+r-2\right)xy^{2}+4\left(pq+pr+qr-2\left(p+q+r\right)+3\right)x^{2}y^{2}\\ & +8\left(2pqr-\left(pq+pr+qr\right)+p+q+r-1\right)x^{2}y^{4}, \end{array}$$

Now from Table 1, Equations (8) and (9)

$$\begin{array}{ll} SSD\left(G\right) & ={\left(S_{y}D_{x}+S_{x}D_{y}\right)\left(f(x,y)\right)|}_{x=y=1}\\ & =20pqr-2\left(pq+pr+qr\right)+4\left(p+q+r\right)-6. \end{array}$$

**Figure 11:**
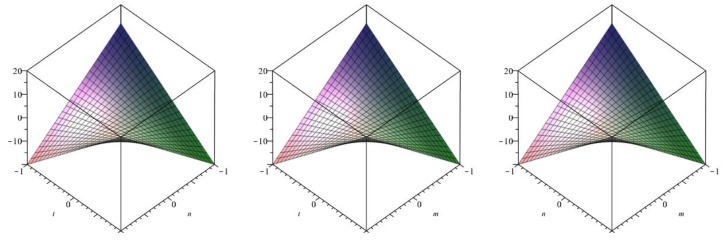
3D plot of Symmetric division index.

The following Figure 11 is a 3D plot of the Symmetric division index (for *p* = 1 left, *q* = 1 middle and *r* = 1 right) and tells us the dependence of the Symmetric division index on the involved parameters. ☐

**Theorem** **8.** *For the graph of the crystallographic structure G* = Cu_2_O[*p,q,r*]*, we have*

$$H\left(G\right)=\frac{8}{3}pqr+\frac{2}{3}\left(pq+pr+qr\right)-\frac{2}{3}.$$

**Proof.**  Let

$$\begin{array}{ll} M\left(G,\;x,y\right)= & 4\left(p+q+r-2\right)xy^{2}+4\left(pq+pr+qr-2\left(p+q+r\right)+3\right)x^{2}y^{2}\\ & +4\left(2pqr-\left(pq+pr+qr\right)+p+q+r-1\right)x^{2}y^{4}. \end{array}$$

Then

(10) $$\begin{array}{ll} S_{x}Jf(x,y)= & \frac{4}{3}\left(p+q+r-2\right)xy^{2}+\left(pq+pr+qr-2\left(p+q+r\right)+3\right)x^{2}y^{2}\\ & +\frac{2}{3}\left(2pqr-\left(pq+pr+qr\right)+p+q+r-1\right)x^{2}y^{4}, \end{array}$$

Now using Table 1 and Equation (10)

$$\begin{array}{ll} H\left(G\right) & =2S_{x}J\left(f(x,y)\right){|}_{x=1}\\ & =\frac{8}{3}pqr+\frac{2}{3}\left(pq+pr+qr\right)-\frac{2}{3}. \end{array}$$

**Figure 12:**
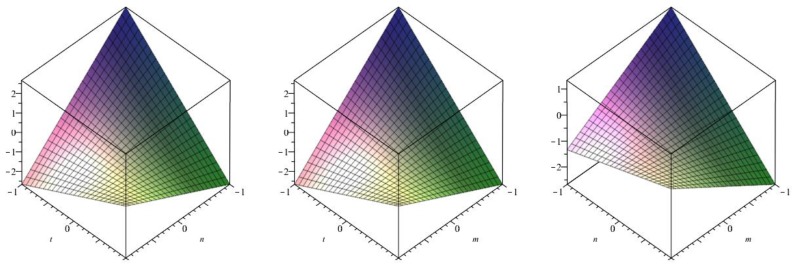
3D plot of Harmonic index.

The following Figure 12 is a 3D plot of the Harmonic index (for *p* = 1 left, *q* = 1 middle and *r* = 1 right) and tells us the dependence of the Harmonic index on the involved parameters. ☐

**Theorem** **9.** *For the graph of crystallographic structure G* = Cu_2_O[*p,q,r*]*, we have*

$$I\left(G\right)=\frac{32}{3}pqr-\frac{4}{3}\left(pq+pr+qr\right)+\frac{4}{3}.$$

**Proof.**  Let

$$\begin{array}{ll} M\left(G,\;x,y\right)= & 4\left(p+q+r-2\right)xy^{2}+4\left(pq+pr+qr-2\left(p+q+r\right)+3\right)x^{2}y^{2}\\ & +4\left(2pqr-\left(pq+pr+qr\right)+p+q+r-1\right)x^{2}y^{4}. \end{array}$$

Then

(11) $$\begin{array}{ll} S_{x}JD_{x}D_{y}f(x,y)= & \frac{8}{3}\left(p+q+r-2\right)xy^{2}+4\left(pq+pr+qr-2\left(p+q+r\right)+3\right)x^{2}y^{2}\\ & +\frac{16}{3}\left(2pqr-\left(pq+pr+qr\right)+p+q+r-1\right)x^{2}y^{4}, \end{array}$$

Now using Table 1 and Equation (11), we get

$$\begin{array}{ll} I\left(G\right) & =S_{x}JD_{x}D_{y}{\left(f(x,y)\right)}_{x=1}\\ & =\frac{32}{3}pqr-\frac{4}{3}\left(pq+pr+qr\right)+\frac{4}{3}. \end{array}$$

**Figure 13:**
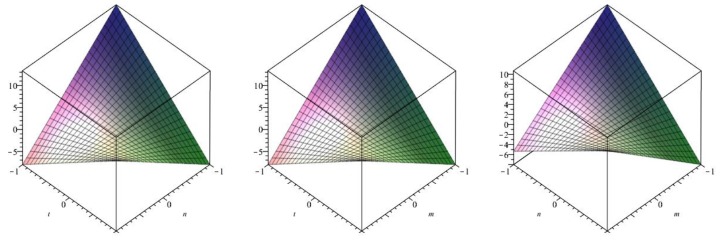
3D plot of Inverse sum index.

The following Figure 13 is a 3D plot of the Inverse sum index (for *p* = 1 left, *q* = 1 middle and *r* = 1 right) and tells us the dependence of the Inverse sum index on the involved parameters. ☐

**Theorem** **10.** *For the graph of the crystallographic structure G* = Cu_2_O[*p,q,r*]*, we have*

A(G)=64pqr.

**Proof.**  Let

$$\begin{array}{ll} M\left(G,\;x,y\right)= & 4\left(p+q+r-2\right)xy^{2}+4\left(pq+pr+qr-2\left(p+q+r\right)+3\right)x^{2}y^{2}\\ & +4\left(2pqr-\left(pq+pr+qr\right)+p+q+r-1\right)x^{2}y^{4}. \end{array}$$

Then

(12) $$\begin{array}{ll} S_{x}{}^{3}Q_{-2}JD_{x}{}^{3}D_{y}{}^{3}f(x,y)= & 32\left(p+q+r-2\right)xy^{2}+32\left(pq+pr+qr-2\left(p+q+r\right)+3\right)x^{2}y^{2}\\ & +\frac{2048}{64}\left(2pqr-\left(pq+pr+qr\right)+p+q+r-1\right)x^{2}y^{4}. \end{array}$$

Using Table 1 and Equation (12), we get

$$\begin{array}{ll} A\left(G\right) & ={S_{x}{}^{3}Q_{-2}JD_{x}{}^{3}D_{y}{}^{3}\left(f(x,y)\right)|}_{x=1}\\ & =64pqr. \end{array}$$

**Figure 14:**
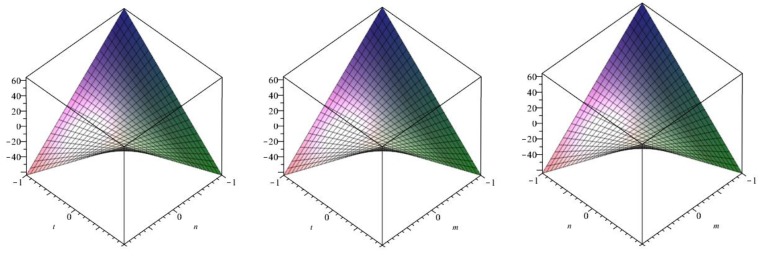
3D plot of Augmented Zagreb index.

The following Figure 14 is a 3D plot of the Augmented Zagreb index (for *p* = 1 left, *q* = 1 middle and *r* = 1 right) and tells us the dependence of the Augmented Zagreb index on the involved parameters. ☐

### 4.2. M Polynomial and Degree-Based Topological Indices for T_i_F_2_[p,q,r]

**Figure 15:**
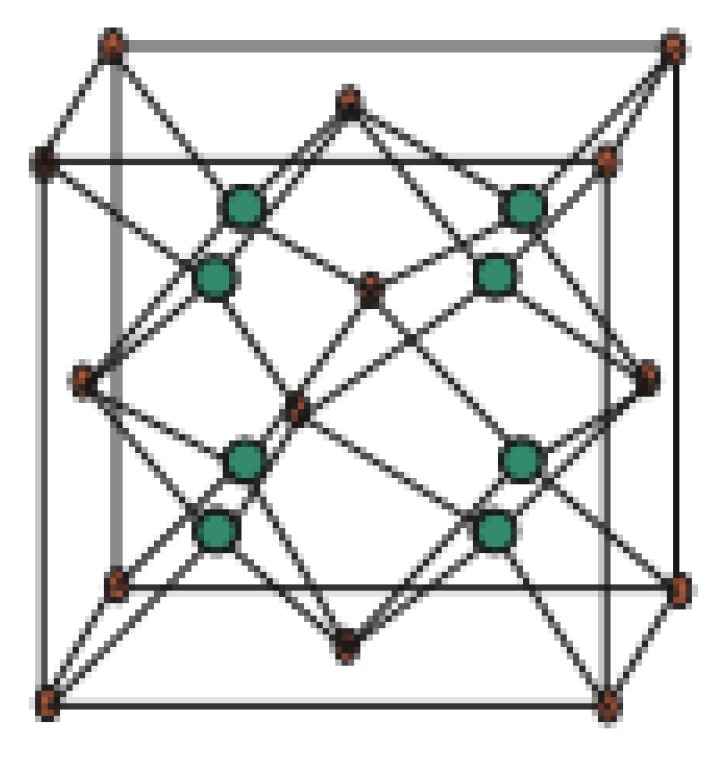
T_i_F_2_[1,1,1].

**Figure 16:**
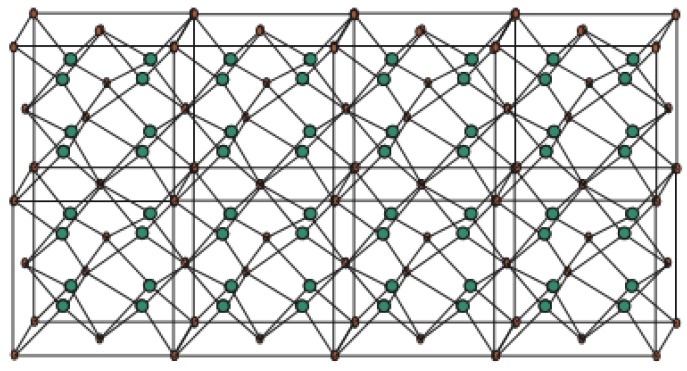
T_i_F_2_[4,1,2].

Titanium difluoride is a water-insoluble titanium hotspot for use in oxygen-delicate applications, for example, metal generation. Fluoride mixes have assorted applications in current advancements and science, from oil refining and carving to engineered natural science and the production of pharmaceuticals. The graph associated with T_i_F_2_*[p,q,r]* is given in Figure 3, see [54]. Let *G* be the chemical graph of T_i_F_2_*[p,q,r]* with p × q unit cells in the plane and *t* layers [23]. In Figure 15 and Figure 16, red dots are for F atoms and green dots are for T_i_ atoms. Now it can be observed easily from Figure 15 and Figure 16 that

$$\left|V\left(T_{i}F_{2}\left[p,q,r\right]\right)\right|=12pqr\;+2pq+2pr\;+2qr\;+\;p\;+\;q\;+\;r\;+1\;\left|E\left(T_{i}F_{2}\left[p,q,r\right]\right)\right|=32pqr.$$

**Theorem** **11.** *For the crystal structure of titanium difluoride G = T_i_F_2_[p,q,r], we have*

$$\begin{array}{ll} M\left(G,\;x,y\right) & =8xy^{4}+8\left(p+q+r-3\right)x^{2}y^{4}+\left[16\left(pq+pr+qr\right)-16\left(p+q+r\right)+24\right]x^{4}y^{4}\\ & +\left[32pqr-16\left(pq+pr+qr\right)+8\left(p+q+r\right)-8\right]x^{4}y^{8}. \end{array}$$

**Proof.**  The edge set of *G*=T_i_F_2_*[p,q,r]* has the following four partitions,

$$E_{1}=E_{\left\{1,4\right\}}=\left\{e=uv\in E\left(G\right)|d_{u}=1,d_{v}=4\right\},$$

$$E_{2}=E_{\left\{2,4\right\}}=\left\{e=uv\in E\left(G\right)|d_{u}=2,\;d_{v}=4\right\},\;E_{3}=E_{\left\{4,4\right\}}=\left\{e=uv\in E\left(G\right)|d_{u}=\;d_{v}=4\right\},\;E_{4}=E_{\left\{4,8\right\}}=\left\{e=uv\in E\left(G\right)|d_{u}=\;4,d_{v}=8\right\},$$

such that

$$\left|E_{1}\left(G\right)\right|=8,$$

$$\left|E_{2}\left(G\right)\right|=8\left(p+q+r-3\right),$$

$$\left|E_{3}\left(G\right)\right|=16\left(pq+pr+qr\right)-16\left(p+q+r\right)+24.\;\left|E_{4}\left(G\right)\right|=32pqr-16\left(pq+pr+qr\right)+8\left(p+q+r\right)-8.$$

Thus the M-polynomial of *T_i_F_2_[p,q,r]* is:

$$\begin{array}{ll} M\left(G;x,y\right) & =\underset{i\leq j}{\sum }m_{ij}\left(G\right)x^{i}y^{j}\\ & =\underset{1\leq 4}{\sum }m_{14}\left(G\right)xy^{4}+\underset{2\leq 4}{\sum }m_{24}\left(G\right)x^{2}y^{4}+\sum_{4\leq 4}m_{44}\left(G\right)x^{4}y^{4}+\sum_{4\leq 8}m_{48}\left(G\right)x^{4}y^{8}\\ & =\underset{uv\in E_{1}}{\sum }m_{14}\left(G\right)xy^{4}+\underset{uv\in E_{2}}{\sum }m_{24}\left(G\right)x^{2}y^{4}+\underset{uv\in E_{3}}{\sum }m_{44}\left(G\right)x^{4}y^{4}+\underset{uv\in E_{4}}{\sum }m_{48}\left(G\right)x^{4}y^{8}\\ & =\left|E_{1}\left(G\right)\right|xy^{4}+\left|E_{2}\left(G\right)\right|x^{2}y^{4}+\left|E_{3}\left(G\right)\right|x^{4}y^{4}+\left|E_{4}\left(G\right)\right|x^{4}y^{8}\\ & =8xy^{4}+8\left(p+q+r-3\right)x^{2}y^{4}+\left[16\left(pq+pr+qr\right)-16\left(p+q+r\right)+24\right]x^{4}y^{4}\\ & +\left[32pqr-16\left(pq+pr+qr\right)+8\left(p+q+r\right)-8\right]x^{4}y^{8}. \end{array}$$

Now we compute some indices by applying fundamental calculus results on the M-polynomial. The proofs of Theorems 12 to 20 are the same as Theorems 2 to 10. ☐

**Theorem** **12.** *For the crystal structure of titanium difluoride G = T_i_F_2_[p,q,r], we have*

$$M_{1}\left(G\right)=384pqr-64\left(pq+pr+qr\right)+16\left(p+q+r\right)-8.$$

**Theorem** **13.** *For the crystal structure of titanium difluoride G = T_i_F_2_[p,q,r], we have*

$$M_{2}\left(G\right)=1024pqr-256\left(pq+pr+qr\right)+64\left(p+q+r\right)-64.$$

**Theorem** **14.** *For the crystal structure of titanium difluoride G = T_i_F_2_[p,q,r], we have*

$${}^{m}M_{2}\left(G\right)=pqr+\frac{1}{2}\left(pq+pr+qr\right)+\frac{1}{4}\left(p+q+r\right)+\frac{1}{4}.$$

**Theorem** **15.** *For the crystal structure of titanium difluoride G = T_i_F_2_[p,q,r], we have*

$$R_{\alpha }\left(G\right)=2^{5\alpha +5}pqr+\left(2^{4\alpha +4}-2^{5\alpha +4}\right)\left(pq+pr+qr\right)+\left(2^{3\alpha +3}-2^{4\alpha +4}+2^{5\alpha +3}\right)\left(p+q+r\right)+\left(2^{2\alpha +3}-3\cdot 2^{3\alpha +3}+3\cdot 2^{4\alpha +3}-2^{5\alpha +3}\right).$$

**Theorem** **16.** *For the crystal structure of titanium difluoride G = T_i_F_2_[p,q,r], we have*

$$RR_{\alpha }\left(G\right)=\frac{1}{2^{5\alpha -5}}pqr+\left(\frac{1}{2^{4\alpha -4}}-\frac{1}{2^{5\alpha -4}}\right)\left(pq+pr+qr\right)+\left(\frac{1}{2^{3\alpha -3}}-\frac{1}{2^{4\alpha -4}}+\frac{1}{2^{5\alpha -3}}\right)\left(p+q+r\right)+\left(\frac{1}{2^{2\alpha -3}}-\frac{3}{2^{3\alpha -3}}+\frac{3}{2^{4\alpha -3}}-\frac{1}{2^{5\alpha -3}}\right).$$

**Theorem** **17.** *For the crystal structure of titanium difluoride G = T_i_F_2_[p,q,r], we have*

SSD(G)=80pqr−8(pq+pr+qr)+8(p+q+r)+2.

**Theorem** **18.** *For the crystal structure of titanium difluoride G = T_i_F_2_[p,q,r], we have*

$$H\left(G\right)=\frac{16}{3}pqr-\frac{4}{3}\left(pq+pr+qr\right)-\frac{2}{15}.$$

**Theorem** **19.** *For the crystal structure of titanium difluoride G = T_i_F_2_[p,q,r], we have*

$$I\left(G\right)=\frac{256}{3}pqr-\frac{32}{3}\left(pq+pr+qr\right)+\frac{16}{15}.$$

**Theorem** **20.** *For the crystal structure of titanium difluoride G = T_i_F_2_[p,q,r], we have*

$$A\left(G\right)=\frac{131072}{125}pqr-\frac{745472}{3375}\left(pq+pr+qr\right)+\frac{76736}{3375}\left(p+q+r\right)+\frac{67264}{3375}.$$

## 5. Concluding Remarks and Discussion

The M-polynomial is interesting, because it helps to compute the first and second Zagreb indices, the modified second Zagreb index, the Randić and Inverse Randić index, the Symmetric division index, the Inverse sum index, the Harmonic index, and the Augmented Zagreb index. Topological indices help us to predict many properties of the understudy molecular compound, for instance the Randić index is a topological descriptor that has been associated with various chemical properties of molecules and has been found to run parallel to preparing the boiling point and Kovats constants of the molecule. The first and second Zagreb indices were found to be useful for finding the total π-electron energy of the molecule. These are among the graph invariants, which were proposed for the estimation of the skeleton of the spreading of the carbon-molecule. Calculation of the distance based topological index of the understudy molecular graphs continues to be a fascinating open problem.

## Figures and Tables

**Table 1:** Derivation of some topological indices from the M-polynomial.

Topological Index	Derivation from M(G;x,y)
First Zagreb	$(D_{x}+D_{y})\left(M\left(G;x,y\right)\right){|}_{x=y=1}$
Second Zagreb	$(D_{x}D_{y})\left(M\left(G;x,y\right)\right){|}_{x=y=1}$
Second Modified Zagreb	$(S_{x}S_{y})\left(M\left(G;x,y\right)\right){|}_{x=y=1}$
$\mathrm{General}\;\mathrm{Randi}\overset{´}{c\;}$	$(D_{x}^{\alpha }D_{y}^{\alpha })\left(M\left(G;x,y\right)\right){|}_{x=y=1}$
General Inverse Randi	$(S_{x}^{\alpha }S_{y}^{\alpha })\left(M\left(G;x,y\right)\right){|}_{x=y=1}$
Symmetric Division Index	$(D_{x}S_{y}+S_{x}D_{y})\left(M\left(G;x,y\right)\right){|}_{x=y=1}$
Harmonic Index	$2\;S_{x}\;J\;{\left(M(G\;;\;x,y)\right)}_{x=1}$
Inverse sum Index	$S_{x}\;J\;D_{x}\;D_{y}{\left(M(G\;;\;x,y)\right)}_{x=1}$
